# Myocardial fibrosis combined with NT-proBNP improves the accuracy of survival prediction in ADHF patients

**DOI:** 10.1186/s12872-021-02083-6

**Published:** 2021-05-28

**Authors:** Yiling Yao, Li Feng, Yanxiang Sun, Shifei Wang, Jie Sun, Bing Hu

**Affiliations:** 1 Department of Cardiology, Zhongshan People Hospital, Sun Yat-Sen University, No. 2 Sunwen East Road, Zhongshan, 528400 Guangdong People’s Republic of China; 2 Department of Cardiology, State Key Laboratory of Organ Failure Research, Nanfang Hospital, Southern Medical University, Guangzhou, People’s Republic of China

**Keywords:** Heart failure, Myocardial fibrosis, Biomarkers, Prognosis

## Abstract

**Background:**

Soluble suppression of tumorigenesis-2 (sST2), Procollagen Type III N-Terminal Peptid (PIIINP) and N-terminal pro-B-type natriuretic peptide (NT-proBNP) have been verified their role in predicting survival in acutely decompensated heart failure (ADHF). However, whether their combination could improve more specific and sensitive prognostic information than NT-proBNP alone remains unclear.

**Methods:**

This was a prospective study, in which 217 ADHF patients at admission were enrolled from November 2018 and August 2019 (mean age 66.18 years ± 13.60, 63.98% male). The blood samples were collected to measure the concentrations of NT-proBNP, sST2 and PIIINP in the first 24 h of hospitalizations. All-cause mortality was registered for all patients after they were discharge over a median period of 339 days.

**Results:**

In univariate Cox analysis, the three biomarkers were predictive of short-term mortality of ADHF patients. After adjusted for some clinical variables including age, admission systolic blood pressure, peripheral edema on admission, history of chronic obstructive pulmonary disease, admission sodium < 135 mmol/L, admission hemoglobin, NT-proBNP, sST2 and PIIINP was significantly associated with the poor outcome (hazard ratio [HR] 1.32, 95% confidence interval [CI] 1.14–1.53, *P* < 0.01; HR 1.21, 95% CI 1.03–1.43, *P* = 0.020; HR 1.40, 95% CI 1.08–1.81, *P* = 0.011). After added with Log2 PIIINP, but not Log2 sST2, the area under the curves (AUC) in the model of clinical variables and Log2 NT-proBNP could increase from 0.79 to 0.85 (95% CI 0.0071–0.10, *P* = 0.024). Furthermore, compared with the model of clinical variables, Log2 NT-proBNP, the improvement in the prognostic model of clinical variables, Log2 NT-proBNP and Log2 PIIINP had statistical significance [net reclassification improvement (NRI) 0.31, *P* = 0.018; integrated discrimination improvement (IDI) 0.068, *P* < 0.01].

**Conclusions:**

NT-proBNP, sST2 and PIIINP are independent prognostic factors for all-cause mortality in ADHF patients. Furthermore, the combination of NT-proBNP and PIIINP may provide incremental prognostic value over NT-proBNP in the survival of ADHF patients.

## Introduction

During the past decade, acutely decompensated heart failure (ADHF), a terminal stage of heart disease characterized by high mortality, has become more and more prevalent, which creates a great burden for health care [1]. Identifying ADHF patients with high mortality risk and implementing effective cardiovascular preventive measures successfully is therefore of great significance for improving those patients’ survival. As one of the remarkable neurohormones from the heart, N-terminal pro-B-type natriuretic peptide (NT-proBNP) is by far an effective indicator that have been verified for its role in predicting the outcomes in both ADHF and chronic heart failure (HF) [2–4], while its specificity and sensitivity remain low somehow [5, 6], which warrants a ADHF predictor with higher specificity and sensitivity.

Myocardial fibrosis, a central role in the formation and development of ADHF, was shown to be associated with cardiovascular outcomes. Furthermore, multiple researches have demonstrated the biomarkers of myocardial fibrosis, such as soluble suppression of tumorigenicity 2 (sST2) and Procollagen Type III N-Terminal Peptid (PIIINP), could serve as predictive factors to identify all-cause death in patients with HF [7–9]. Theoretically, heavier myocardial fibrosis in patients with heart failure means more severe ventricular remodeling of patients. Moreover, severe ventricular remodeling usually associates with poor outcomes in ADHF patients. Thus, it is possible that NT-proBNP combining with biomarkers of myocardial fibrosis would improve the accuracy of NT-proBNP in predicting major adverse outcomes [10]. However, no research before has focused on the combined value of sST2, PIIINP and NT-proBNP in identifying all-cause death in ADHF patients.

The current study was aimed to investigate whether the combination of sST2, PIIINP and NT-proBNP was more accurate than NT-proBNP alone in predicting short-term survival in patients with ADHF.

## Methods

### Study population and design

Between November 2018 and August 2019, 217 consecutive patients with ADHF were enrolled prospectively in Zhongshan People's Hospital, Guangdong Province, China. According to the related guidelines [2, 3], those patients in the study should have the signs and symptoms which indicated they had an attack of new-onset HF or acutely decompensated chronic HF (e.g., dyspnea, edema, weight gain). What’s more, they should be observed with elevated levels of NT-proBNP and the impaired systolic or diastolic function of heart by echocardiography. Each patient’ clinical data would be collected by two cardiologists within the first 24 h of the admission, including age, gender, body-mass index (BMI), systolic blood pressure (SBP), heart rate, comorbidities and New York Heart Association (NYHA) class. Furthermore, the results of laboratory examination such as Hemoglobin (Hb), Serum Sodium, Serum Creatinine (Scr), Low-Density Lipoprotein Cholesterol (LDL-c), Hemoglobin A1c (HbA1c) and Procalcitonin (PCT) would be recorded; and Left ventricular ejection fraction (LVEF) was tested with Simpson´s method. The blood sample would be collected within the first 24 h when they were on admission for the measurement of the levels of NT-proBNP, sST2 and PIIINP. In order to avoid the interferences in the biomarkers of myocardial fibrosis concentrations from other diseases, those who presented severe extracardial condition would not be included such as pulmonary interstitial fibrosis, metabolic bone diseases, liver diseases, or renal failure. Other exclusion criteria were age < 18 years, acute coronary syndrome, unstable hemodynamics, cardiac surgery and malignant tumor.

The follow-up period would start from the first day when participants were discharged. The subjects would be investigated by the cardiologists who did not know about the results of the biomarkers. All the patients were treated with standard HF management according to the current clinical practice guidelines, and no one was observed to quit the study during the follow-up period. Data of all-cause mortality was collected with the means of regular telephone interviews, administrative databases and medical records monthly.

The study was performed in accordance with the Declaration of Helsinki, and approved by the local Regional Ethics Committee. All the participants in the study had signed written informed consent already.

### Biochemical measurement

Blood samples would be centrifuged at + 4 °C after collection immediately and stored at − 80 °C at the laboratory until analysis. Serum sST2 were measured with human ST2 Quantikine ELISA Kit (R&D Systems, Inc., Minneapolis, Minnesota, United States), in which the lower limit of detection is 0.005 ng/ml and measurement range is up to 2 ng/ml. The inter- and intra-assay coefficients of variation were 4.4–5.6% and 5.4–7.1%, respectively [11]. Now the risk threshold of sST2 in predicting all-cause death is still not validated enough accurately, and the range between 35 and 65 ng/ml was proposed by some studies according to Youden Index [12–15]. Serum PIIINP was measured with the Chemiluminescent Microparticle Immunoassay (AnTu Biological Co., Zhengzhou, China). The range of detection is 3.0–100.0 ng/ml. The coefficients of variation were < 15.0%. Serum NT-proBNP was tested by electrochemiluminescence immunoassay (Roche, Basel, Switzerland), and the upper limit of normal for NT-proBNP in the acute setting is < 300 pg/ml.

### Statistical analysis

The median ± standard deviation or median and interquartile range was presented to describe the basic characters for continuous variables, and the percentage for categorical variables. For those continuous variables with a normal distribution and equal variance, the independent-sample student’s T-test or ANOVA was used to compare the differences of two groups. Otherwise, rank sum test was used. *Chi*-*square* tests were chosen to evaluate the statistic significances between different groups in categorical variables. During the follow-up, all-cause death in ADHF patients was seen as the endpoint in the study.

2Log transformations were made for the three biomarkers in the following analysis because of their skewed distribution. Cox proportional hazards regression was conducted to perform the association between the biomarkers and mortality in ADHF patients. Univariable Cox regression analysis would study weather NT-proBNP, sST2, PIIINP and other clinical variables were related to the all-cause death of ADHF patients, respectively. Adjusted by several clinical variables with utility of prognostic value in ADHF patients, the three biomarkers above were evaluated in the bootstrapped multivariable Cox regression analysis with Stepwise method. The 2log-transformed biomarkers with prognostic information would be added into clinical variables only model to build new models. Built the receiver operating characteristic (ROC) curves of the models, and calculated the area under the curves (AUC), respectively. The comparisons between models by a pair-wise method were performed with the method of Hanley JA and McNeil BJ [16]. What’s more, net reclassification improvement (NRI) and absolute integrated discrimination index (IDI) would be tested to evaluate the improvement in predictive accuracy between the models by a pair-wise method.

When a *P*-value < 0.05, it was considered statistically significant. PASW statistics version 24.0 (SPSS Inc., Chicago, IL, USA), MedCalc statistical software version 19.0.7 and R version 4.0.2 (http://www.r-project.org) were used to analyze the data attained in the study.

## Results

### Patient characteristics

The baseline characteristics of ADHF patients enrolled were showed in Table 1. The mean age of subjects was 66.18 ± 13.60 years and male 64.98%. 167 subjects (76.96%) had NYHA class III or IV prior to decompensation and 127 subjects (58.53%) peripheral edema on admission. The mean LVEF was 45.00% (32.00, 58.75). Comorbidities were: hypertension (57.14%), coronary artery disease (45.16%), atrial fibrillation (39.17%), *Diabetes mellitus* (27.65%) and chronic obstructive pulmonary disease (10.60%). The mean concentration of sST2 is 19.63 ng/ml (12.61, 35.53), PIIINP 6.70 ng/ml (4.84, 9.96), and NT-proBNP 4491.00 pg/ml (1814.50, 8436.00). While the ADHF patients were treated with heart failure management, 84.79% of them used angiotensin-converting enzyme inhibitor or angiotensin-receptor blocker, 72.35% β- blocker, 93.09% Loop diuretic, and 87.10% Aldosterone antagonist.

**Table 1:** Baseline characteristics of ADHF patients (n = 217)

Age (years)	66.18 ± 13.60
Male, n (%)	141 (64.98)
BMI (kg/m^2^)	24.82 ± 3.65
SBP (mmHg)	141.22 ± 24.99
Heart rate (bmp)	91.61 ± 23.46
Prior NYHA class I–II/III–IV, n (%)	50/167 (23.04/76.96)
Peripheral edema, n (%)	127 (58.53)
Hypertension, n (%)	124 (57.14)
CAD, n (%)	98 (45.16)
AF, n (%)	85 (39.17)
*Diabetes mellitus*, n (%)	60 (27.65)
COPD, n (%)	23 (10.60)
Hemoglobin (g/dl)	12.69 ± 2.18
Sodium < 135 mmol/L, n (%)	14 (6.45)
SCr (umol/L)	94.00 (79.00, 120.50)
LDL-c (mmol/L)	2.45 (1.73, 3.15)
HbA1c (%)	6.00 (5.70, 7.00)
PCT (pg/ml)	0.064 (0.038, 0.12)
sST2 (ng/ml)	19.63 (12.61, 35.53)
PIIINP (ng/ml)	6.70 (4.84, 9.96)
NT-proBNP (pg/ml)	4491.00 (1814.50, 8436.00)
LVEF (%)	45.00 (32.00, 58.75)
ACEI/ARB, n (%)	184 (84.79)
β-blocker, n (%)	157 (72.35)
Loop diuretic, n (%)	202 (93.09)
Aldosterone antagonist, n (%)	189 (87.10)

ACEI, angiotensin-converting enzyme inhibitor; AF, atrial fibrillation; ARB, angiotensin-receptor blocker; BMI, body-mass index; CAD, coronary artery disease; COPD, chronic obstructive pulmonary disease; HbA1c, Hemoglobin A1c; LDL-c, low-density lipoprotein cholesterol; LVEF, left ventricular ejection fraction; NT-proBNP, N-terminal pro B-type natriuretic peptide; NYHA, New York Heart Association; PCT, procalcitonin; PIIINP, procollagen type III N-terminal peptid; SBP, systolic blood pressure; SCr, serum creatinine; sST2, soluble suppression of tumorigenicity-2

### The biomarkers and the results of the survival analysis for all-cause mortality in ADHF patients

37 patients (17.05%) had died during the follow-up (median follow-up period was 339 days [249, 402]). Compared with those alive at discharge, patients who died after going home from hospital were older, and the levels of admission NYHA class were higher; however, LVEF between two groups was of no statistical significance [50.00 (32.50, 56.00) vs 44.00 (32.00, 60.00), *P* = 0.91]. The levels of NT-proBNP, sST2, and PIIINP among the population that died are higher than the others (pg/ml, 8481.00 (5064.00, 13,882.50) vs 3947.50 (1644.75, 7580.50), *P* < 0.01; ng/ml, 28.56 (15.91, 61.22) vs 19.03 (11.91, 32.12), *P* = 0.010; ng/ml, 9.22 (5.00, 11.68) vs 6.14 (4.55, 8.71), *P* < 0.01). For *Diabetes* patients, those who did not survive had higher concentration of the NT-proBNP compared with those alive [10124.50 (4211.50, 20,064.00) vs 3330.50 (1682.25, 6547.75), *P* = 0.013].

The Univariate Cox regression analysis showed sST2, PIIINP and NT-proBNP to be predictive of all-cause mortality (*P* < 0.01, respectively) (Table 2). Some clinical variables have been validated with their prognostic utility for all-cause mortality of patients with ADHF; therefore, they were made to a clinical adjustment model for determining if the 3 biomarkers had an independent value [age (per 10 years), admission systolic blood pressure (per 10 mm Hg), peripheral edema on admission, history of chronic obstructive pulmonary disease, admission sodium < 135 mmol/L, admission hemoglobin] [17–19]. After adjusted with the clinical variables above, the results from bootstrapped multivariate Cox regression analysis indicated that sST2, PIIINP and NT-proBNP were the prognostic factors of all-cause mortality independently (HR_2log_ 1.21, 95% CI 1.03–1.43, *P* = 0.020; HR_2log_ 1.40, 95% CI 1.08–1.81, *P* = 0.011; HR_2log_ 1.32, 95% CI 1.14–1.53, *P* < 0.01) (Table 2).

**Table 2:** Univariate and bootstrapped multivariate Cox regression analysis for all-cause mortality

Variables	Univariable		Multivariate	
	HR (95% CI)	*P*	HR (95% CI)	*P*
Log2 sST2	1.23 (1.06–1.44)	**< 0.01**	1.21 (1.03–1.43)	**0.020**
Log2 PIIINP	1.38 (1.11–1.71)	**< 0.01**	1.40 (1.08–1.81)	**0.011**
Log2 NT-proBNP	1.34 (1.17–1.54)	**< 0.01**	1.32 (1.14–1.53)	**< 0.01**

Bold indicates the siginificant values (*P* < 0.05)

HR, hazard rate; CI, confidence interval

Model 1 consisted of the six clinical factors above. Model 2 was made up of Model 1 and Log2 NT-proBNP. Model 3 was constructed by adding Model 2 and Log2 sST2, and Model 4 by adding Model 2 and Log2 PIIINP. Model 5 was built by combining Model 2 with Log2 sST2 and Log2 PIIINP. Table 3 showed the ROC curves of models for predicting the all-cause mortality of ADHF patients, in which the AUC of Model 1, 2, 3, 4 and 5 were 0.77 (95% CI 0.69–0.84), 0.79 (95% CI 0.71–0.86), 0.80 (95% CI 0.72–0.86), 0.85 (95% CI 0.77–0.90) and 0.85 (95% CI 0.77–0.90), respectively (all *P* < 0.01). Among them, the area under the curves of Model 4 performed better than that of clinical variables only model (Model 1), Model 2 and Model 3, respectively (ΔAUC 0.076, 95% CI 0.0044–0.15, *P* = 0.038; ΔAUC 0.054, 95% CI 0.0071–0.10, *P* = 0.024; ΔAUC 0.050, 95% CI 0.00048–0.099, *P* = 0.048) (Table 4). More importantly, the comparisons of the area under the curves between Model 5, which contained additional Log2 sST2 compared with Model 4, and the three models above also got statistically significant results; however, Model 4 and Model 5 performed so similar that they could be approximately the same in predicting the death in ADHF patients (ΔAUC < 0.0010, *P* = 1.00) (Table 4). Besides, for ADHF patients, the prognosis of all-cause mortality had made no significant difference between Model 3 added with Log_sST2 and Model 2 added with Log2 NT-proBNP (ΔAUC 0.016, 95% CI − 0.011 to 0.043, *P* = 0.25) (Table 4). More details about the different ROC curves of models were shown in Table 4.

**Table 3:** The AUC of the ROC curves from different models and the comparisons

	AUC	95% CI	*P*	Sensitivity	Specificity
Model 1	0.77	0.69–0.84	**< 0.01**	0.69	0.75
Model 2	0.79	0.71–0.86	**< 0.01**	0.61	0.87
Model 3	0.80	0.72–0.86	**< 0.01**	0.92	0.61
Model 4	0.85	0.77–0.90	**< 0.01**	0.94	0.63
Model 5	0.85	0.77–0.90	**< 0.01**	0.94	0.63

Bold indicates the siginificant values (*P* < 0.05)

Model 1: Clinical factors contained age, gender, NYHA class, AF, SCr and LVEF; Model 2: Model 1 + Log2 NT-proBNP; Model 3: Model 2 + Log2 sST2; Model 4: Model 2 + Log2 PIIINP; Model 5: Model 2 + Log2 sST2 + Log2 PIIINP

AUC, area under curve; CI, confidence interval

**Table 4:** The comparisons of the ROC curves between two different models

	△AUC	95% CI	*P*	△AUC	95% CI	*P*
Compared to Model 1				Compared to Model 2		
Model 2	0.060	− 0.022 to 0.14	0.15	–	–	–
Model 3	0.073	− 0.0082 to 0.16	0.078	0.016	− 0.011 to 0.043	0.25
Model 4	0.076	0.0044–0.15	**0.038**	0.054	0.0071–0.10	**0.024**
Model 5	0.076	0.0044–0.15	**0.038**	0.054	0.0071–0.10	**0.024**
Compared to Model 3				Compared to Model 4		
Model 4	0.050	0.00048–0.099	**0.048**	–	–	–
Model 5	0.050	0.00048–0.099	**0.048**	0.00	–	1.0

Bold indicates the siginificant values (*P* < 0.05)

Model 1: Clinical factors contained age, gender, NYHA class, AF, SCr and LVEF; Model 2: Model 1 + Log2 NT-proBNP; Model 3: Model 2 + Log2 sST2; Model 4: Model 2 + Log2 PIIINP; Model 5: Model 2 + Log2 sST2 + Log2 PIIINP

CI, confidence interval

NRI and IDI were used to evaluate the improvement of predictive Models for all-cause mortality in ADHF patients (Table 5). Adding Log2 PIIINP instead of Log2 sST2 to the model of clinical risk factors and Log2 NT-proBNP (Model 2) and forming Model 4 could make the NRI improve 0.31 (95% CI 0.053–0.56, *P* = 0.018) and IDI improve 0.068 (95% CI 0.024–0.11, *P* < 0.01). Simultaneously, the improvement of NRI and IDI would be validated in the setting of Model 5 in which Log2 PIIINP and Log2 sST2 were added to Model 2 (NRI: 0.31 improvement, *P* = 0.018; IDI: 0.068 improvement, *P* < 0.01). It was worth noting that there was no statistically improvement in neither NRI nor IDI when Model 5 was compared with Model 4, even though there was the additional independent predictor Log2 sST2 in Model 5. The results above suggested that combining with PIIINP could make the model of NT-proBNP and clinical variables predict ADHF patients’ mortality better. Remarkably, the addition of PIIINP and sST2 did not perform better than the addition of PIIINP alone for the model of NT-proBNP and clinical variables in predicting ADHF patients’ death.

**Table 5:** Predictive improvement in the models for all-cause mortality

Model	NRI	95% CI	*P*	IDI	95% CI	*P*
Model 2	0.68*	0.36–1.01	**< 0.01**	0.12*	0.056–0.18	**< 0.01**
Model 3	0.64*	0.31–0.97	**< 0.01**	0.14*	0.072–0.21	**< 0.01**
	0.10**	− 0.25 to 0.46	0.56	0.021**	− 0.0042 to 0.046	0.10
Model 4	0.52*	0.20–0.84	**< 0.01**	0.17*	0.10–0.24	**< 0.01**
	0.31**	0.053–0.56	**0.018**	0.068**	0.024–0.11	**< 0.01**
	0.20***	− 0.12 to 0.52	0.23	0.054***	0.0065–0.10	**0.026**
Model 5	0.52*	0.20–0.84	**< 0.01**	0.17*	0.10–0.24	**< 0.01**
	0.31**	0.053–0.56	**0.018**	0.068**	0.024–0.11	**< 0.01**
	0.20***	− 0.12 to 0.52	0.23	0.054***	0.0068–0.10	**0.025**
	0.011****	− 0.36 to 0.40	0.95	0.00	–	1.0

Bold indicates the siginificant values (*P* < 0.05)

Model 1: Clinical factors contained age, gender, NYHA class, AF, SCr and LVEF; Model 2: Model 1 + Log2 NT-proBNP; Model 3: Model 2 + Log2 sST2; Model 4: Model 2 + Log2 PIIINP; Model 5: Model 2 + Log2 sST2 + Log2 PIIINP

CI, confidence interval; NRI, net reclassification improvement; IDI, integrated discrimination improvement

^*^Compared with Model 1; **compared with Model 2;***compared with Model 3; ****compared with Model 4

## Discussion

The current study found sST2, PIIINP and NT-proBNP were respectively three independent risk factors for short-term all-cause death in patients with ADHF, and higher levels of these biomarkers were associated with higher risk of all-cause death. More importantly, PIIINP could improve risk stratification with NT-proBNP in predicting short-term survival among ADHF patients.

Multiple studies have shown higher NT-proBNP level was associated with higher incidence of poor clinical outcomes, including heart failure readmission, cardiovascular mortality, all-cause mortality, and composite outcomes in ADHF patients. Data from Mario Petretta et al. demonstrated that NT-proBNP levels may differentiate chronic heart failure patients with a higher risk of cardiac mortality [4]. The study from Santaguida PL et al. showed that NT-proBNP was an independent predictor of all-cause and cardiovascular mortality in ADHF [20–22]. In addition, Savarese G and the colleagues found that higher level of NT-proBNP could predict higher risk of HF-related admission [23]. Based on these studies, NT-proBNP was recommended by current guidelines as a prognostic marker in ADHF patients [2, 3]. *Diabetics* was demonstrated as the risk factor to affect clinical outcomes in HF [24], and among them the high concentrations of NT-proBNP was associated with high rate of rehospitalization for acutely heart failure [24]. Consistently, the current study found that ADHF patients with increased NT-proBNP level were associated with worse outcomes than those without. However, caution should be noted that the level of the biomarker in patients could be influenced by a wide variety of factors including age, weight, renal function, and noncardiac diseases [5, 6]. Simultaneously, our study have demonstrated that NT-proBNP could not provide incremental prognostic value significantly after added to clinical variables only model according to the comparison of AUC between two models (Model 1 and Model 2) in the study, indicating that strategies combining other biomarkers may inform clinicians to make further NT-proBNP-based risk stratification in ADHF patients.

PIIINP, one of the biomarkers of myocardial fibrosis studied in the current study, was observed to be deposited in cardiac extracellular matrix during the development of cardiac remodeling, resulting in worse ventricular compliance as well as poor outcomes [25]. In fact, PIIINP could also be regarded as the surrogate of myocardial fibrosis. It was reported that high level of circulating PIIINP was predictive of increased all-cause mortality and HF readmission risk among African Americans with chronic HF [9]. Similarly, the data shown in the study demonstrated that high level of PIIINP was strongly linked to the incidence of all-cause death in patients with ADHF, no matter adjusting or not adjusting confounding factors, further suggesting that it was an emerging prognostic factors independently for all-cause mortality among ADHF patients.

Considering this, the current study firstly combined NT-proBNP with PIIINP and intended to improve the accuracy of survival prediction in ADHF patients. Our data showed the combination of NT-proBNP and PIIINP was significantly more effective than NT-proBNP alone in predicting all-cause death in patients with ADHF. After adding Log2 PIIINP to the model of Log2 NT-proBNP and clinical variables, the sensitivity of the new model (Model 4) in predicting ADHF patients’ survival could be up to 0.94, which would invariably accord with the statistically significant improvement of AUC, NRI, and IDI. In addition to NT-proBNP, the data comparing the combination of two biomarkers generated from different pathological mechanism (NT-proBNP and PIIINP) and NT-proBNP alone have demonstrated the role of PIIINP in ADHF patients screening to detect those who would survive in the short-term follow-up should be valued in clinical practice. In fact, with regard to the reason for the increased prognostic information brought by PIIINP on the basis of NT-proBNP in ADHF patients, the severity of HF symptoms and myocardial remodeling should be focused on. Theoretically, ADHF patients with poor survival usually accompany with increased levels of myocardial remodeling and fibrosis. In other words, with low level of myocardial fibrosis, those patients are more likely to survive during the follow-up. Therefore, as one of biomarkers of myocardial fibrosis, low PIIINP values are linked to early stage of myocardial and matrix remodeling and, sometimes possibly, means the cardiac remodeling has not even existed in HF progression, resulting in an efficient cardiac function and an acceptable clinical outcome in ADHF patients. Notably, our predictive model of NT-proBNP combined with PIIINP not only took into account the disorder of cardiac neuroendocrine function in patients with ADHF, but also considered the structural disorders of cardiac remodeling, thus was more sensitive than NT-proBNP alone in predicting short-term all-cause mortality. In short, the current study firstly came out with a survival model of NT-proBNP combined with PIIINP, and found that the combination of the two biomarkers could perform better than NT-proBNP alone to rule out for the prognosis of short-term all-cause mortality in ADHF patients.

sST2 is a common biomarker of myocardial fibrosis, whose increase was associated with higher risks of HF adverse events [26–28]. Previous researches have demonstrated the predictive value of sST2 for HF patients, in which ADHF patients with higher level of admission sST2 suffered higher risk of all-cause mortality and cardiovascular death than those without. The current study added some new evidence into this area by demonstrating that circulating sST2 value in ADHF patients could be a strong independent predictor of short term all-cause death. However, compared with PIIINP, sST2 showed nonsignificant incremental information on the basis of prognostic model of NT-proBNP and clinical variables in patients with ADHF. In fact, although both sST2 and PIIINP could be used to evaluate the degree of myocardial remodeling, the former awaits more available clinical evidence to validate the association between it and myocardial fibrosis histologically compared to the latter [25, 29, 30], which may indicate the biomarker is less accurate than PIIINP in assessing myocardial remodeling in ADHF patients. In fact, as the members of the IL-1 receptor-like family of proteins, the resource of sST2 is still indefinite [31–33]. In the study, the sST2 values could not provide more prognostic information after adding to the model of NT-proBNP, PIIINP and clinical variables than before in ADHF patients’ survival, suggesting that the combination of NT-proBNP and two biomarkers of fibrosis may not be more effective than that of NT-proBNP and PIIINP.

However, several limitations should be noted when interpreting. Firstly, the current study was performed on single center with relative small sample size, the conclusion of which still warrants confirmed in multiple centers with larger sample size. Nevertheless, the current study has validated the models built by bootstrapping internally. Besides, the multi-biomarker approach consistently showed a positive result in model performance when considering confounding factors of clinical variables, further demonstrating a survival diagnosis improvement. Secondly, previous studies found that changes of NT-proBNP, sST2 and PIIINP levels during follow-up were also shown to be associated with the prognosis of HF respectively [34, 35], while the changes were not considered in this study because all the circulating biomarkers were tested only once when discharged.

## Conclusions

The current study suggested that high levels of NT-proBNP, sST2 and PIIINP were associated with increased mortality in ADHF patients. Furthermore, PIIINP could take complementary role for risk stratification in all-cause mortality among ADHF patients next to NT-proBNP.

## Data Availability

The datasets generated and analyzed during the current study are not publicly available due to the consent given by study participants but are available from the corresponding author on reasonable request.

## Abbreviations

ACEI: Angiotensin-converting enzyme inhibitor
ADHF: Acutely decompensated heart failure
ARB: Angiotensin-receptor blocker
AUC: Area under the curves
BMI: Body-mass index
CI: Confidence interval
COPD: Chronic obstructive pulmonary disease
Hb: Hemoglobin
HbA1c: Hemoglobin A1c
HF: Heart failure
HR: Hazard ratio
IDI: Integrated discrimination index
LDL-c: Low-density lipoprotein cholesterol
LVEF: Left ventricular ejection fraction
NT-proBNP: N-terminal pro-B-type natriuretic peptide
NRI: Net reclassification improvement
NYHA: New York Heart Association
PCT: Procalcitonin
PIIINP: Procollagen type III N-terminal peptid
SBP: Systolic blood pressure
SCr: Serum creatinine
sST2: Soluble suppression of tumorigenicity-2
